# Labeling of Mucin-Type *O*-Glycans for Quantification Using Liquid Chromatography and Fluorescence Detection

**DOI:** 10.1021/acsmeasuresciau.3c00071

**Published:** 2024-02-14

**Authors:** Marc Safferthal, Leïla Bechtella, Andreas Zappe, Gaël M. Vos, Kevin Pagel

**Affiliations:** † Fritz Haber Institute of the Max Planck Society, Faradayweg 4-6, 14195 Berlin, Germany; ‡ Department of Biology, Chemistry, Pharmacy, Freie Universität Berlin, Altensteinstraße 23a, 14195 Berlin, Germany

**Keywords:** mucin-type *O*-glycans, labeling, quantification, fluorescence detection, hydrophilic interaction liquid chromatography, mass spectrometry

## Abstract

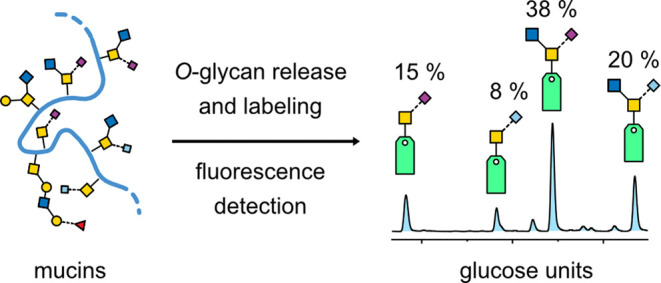

*O*-glycosylation is a common post-translational modification that is essential for the defensive properties of mucus barriers. Incomplete and altered *O*-glycosylation is often linked to severe diseases, such as cancer, cystic fibrosis, and chronic obstructive pulmonary disease. Originating from a nontemplate-driven biosynthesis, mucin-type *O*-glycan structures are very complex. They are often present as heterogeneous mixtures containing multiple isomers. Therefore, the analysis of complex *O*-glycan mixtures usually requires hyphenation of orthogonal techniques such as liquid chromatography (LC), ion mobility spectrometry, and mass spectrometry (MS). However, MS-based techniques are mainly qualitative. Moreover, LC separation of *O*-glycans often lacks reproducibility and requires sophisticated data treatment and analysis. Here we present a mucin-type *O*-glycomics analysis workflow that utilizes hydrophilic interaction liquid chromatography for separation and fluorescence labeling for detection and quantification. In combination with mass spectrometry, a detailed analysis on the relative abundance of specific mucin-type *O*-glycan compositions and features, such as fucose, sialic acids, and sulfates, is performed. Furthermore, the average number of monosaccharide units of *O*-glycans in different samples was determined. To demonstrate universal applicability, the method was tested on mucins from different tissue types and mammals, such as bovine submaxillary mucins, porcine gastric mucins, and human milk mucins. To account for day-to-day retention time shifts in *O*-glycan separations and increase the comparability between different instruments and laboratories, we included fluorescently labeled dextran ladders in our workflow. In addition, we set up a library of glucose unit values for all identified *O*-glycans, which can be used to simplify the identification process of glycans in future analyses.

## Introduction

*N*- and *O*-linked glycosylations are among the most common but also most complex post-translational modifications of proteins. It is estimated that more than 70% of eukaryotic and 50–70% of human proteins are glycosylated.^1−3^ The occurrence of severe diseases like cancer, chronic obstructive pulmonary disease, and cystic fibrosis is often associated with incomplete or altered glycosylation.^4,5^ Thus, structural analysis of glycans as biomarkers is receiving increasing attention. Although *N*- and *O*-glycans are often found on the same proteins, they differ strongly in their compositions and biosynthesis.

All *N*-glycans are extended from a common trimannosyl chitobiose core structure (Man_3_GlcNAc_2_) and can be detached from the protein backbone by enzymes such as peptide:*N*-glycosidase F (PNGase F). Commercial availability of this universal enzyme enabled the development of simple and efficient methods for *N*-glycan analysis in the past. In standard *N*-glycan analysis workflows, the released glycans are tagged by reductive amination with fluorescence labels such as aminobenzoic acid (AA), aminobenzamide (AB), and procainamide (ProA).^6−8^ These labels have been shown to increase the ionization efficiency in mass spectrometry (MS) and allow the fluorescence detection (FLD) of *N*-glycans. Due to the selective attachment of one label per glycan, fluorescence labeling further allows for direct quantification by FLD. The labeled *N*-glycans are commonly separated based on their polarity using hydrophilic interaction liquid chromatography (HILIC) and detected by FLD and MS.^7^ High-throughput *N*-glycan analysis often makes use of depolymerized and labeled dextran (dextran ladder), which is used to calibrate the glycan retention times. This ensures reproducibility and enables interlaboratory comparison.^9,10^ Typically, the retention times are converted into glucose units (GU) by comparing them to the retention times of the dextran ladder oligosaccharides. GU values can be embedded into databases and used for the assignment of complex *N*-glycan structures.^10,11^ Ion mobility spectrometry (IMS) is often used in addition to HILIC as it enables rapid separation of glycan isomers in the gas phase.^12−14^ Comparable with GUs, the drift times of glycans are converted into collision cross sections (CCS), which can be stored in databases to facilitate the structural assignment of glycans.^15,16^

Mucin-type *O*-glycans are commonly found on mucins, a class of densely glycosylated, high molecular weight proteins (>200 kDa). Mucins can be secreted as a primary component of mucus or membrane-bound as part of the glycocalyx. Initiated by an *N*-acetylgalactosamine (GalNAc) unit, mucin-type *O*-glycans are based on one of eight core structures, which are extended by *N*-acetyllactosamine repeating units, terminated by fucose and sialic acids, and decorated with sulfates and different blood group epitopes.^17^ Specific glycosyltransferases attach monosaccharides to different positions of the *O*-glycan core structures, which leads to the frequent coexistence of multiple isomers. The resulting structural diversity and occurrence of isomers represent fundamental challenges for their analysis.^18^

The major limitation in *O*-glycomics is that no universal enzyme, which is capable of cleaving all intact *O*-glycan core structures simultaneously, has been identified to date. A common alternative is rather harsh chemical release methods based on oxidation,^19,20^ alkaline β-elimination,^21,22^ and hydrazinolysis.^23^ These methods enable the efficient release of *O*-glycans, albeit at the cost of a hydrolyzed protein backbone. The chemical release of *O*-glycans by β-elimination remains the most widely used method to date. In this method, *O*-glycans are detached from the protein backbone and reduced to alditols. After purification and desalting steps involving solid-phase extraction (SPE), the *O*-glycan alditol mixtures are analyzed using liquid chromatography coupled to tandem mass spectrometry (LC–MS/MS). Traditionally, the glycan isomers are separated by porous graphitized carbon (PGC) chromatography and fragmented by collision-induced dissociation (CID). The resulting tandem mass spectra contain cross-ring fragments diagnostic to *O*-glycan branching and regiochemistry.^22,24−26^ Specialized databases can be used to simplify the identification of glycans.^27^ However, the identification of glycan structures remains laborious and prone to error. Recently, IMS was introduced to the field of *O*-glycomics, which has shown great potential for the gas-phase separation and identification of complex *O*-glycan isomers.^28,29^

MS-based methods have proven their efficiency for general *O*-glycan profiling and structural identification. However, they are often purely qualitative because the abundance of specific glycan signals is linked to not only their concentration but also their ionization efficiency and the sample matrix. Therefore, MS-based techniques are only semiquantitative, and methods for direct quantification of *O*-glycans are urgently needed to gain a better understanding of the underlying biological *O*-glycosylation processes, which are important in studying disease progression.^5,30^ While the use of fluorescence labeling and FLD is a standard technique in *N*-glycomics,^31−37^ examples for *O*-glycan analysis remain scarce. First efforts linking fluorescence labeling to *O*-glycan analysis have been described recently.^38−41^ However, a complete study demonstrating its potential for the quantification of complex mucin-type *O*-glycan samples is still lacking.

In this study, we developed an analytical workflow for the release, fluorescence labeling, separation, compositional analysis, and quantification of mucin-type *O*-glycans. The method was applied to map common *O*-glycan features in heavily *O*-glycosylated mucins from human, bovine, and porcine origin. In addition, a dextran ladder was included in the workflow, from which a library of GU values was compiled.

## Materials and Methods

### Chemicals

HPLC-grade acetonitrile (ACN), methanol (MeOH), acetone, and trifluoroacetic acid (TFA) were purchased from VWR Chemicals. Porcine gastric mucins (PGM) type III, bovine submaxillary mucins (BSM) Type I-S, fetuin from fetal bovine serum, dextran *M*_w_ 1000, dimethylformamide (DMF), dimethyl sulfoxide (DMSO), ammonium acetate, and Discovery Glycan SPE tubes were obtained from Sigma-Aldrich. Procainamide hydrochloride and Hypersep Hypercarb SPE tubes (50 mg) were purchased from Thermo Fisher Scientific. Sodium cyanoborohydride was obtained from abcr GmbH (Germany). Human milk fat globule was obtained from AMMEVA GmbH (Germany).

### Purification of Mucins from Human Milk Fat Globule

Human milk fat globule (13.6 g) was dissolved in 30 mL of CHCl_3_:MeOH (2:1) and the mixture was put under agitation. After 5 min of agitation, the mixture was centrifuged for 15 min at 4 °C, and the aqueous phase was collected. Ten mL of H_2_O was added to the organic phase and the mixture was agitated for 5 min before centrifugation for 15 min at 4 °C. Aqueous phases were combined and washed with 30 mL of CHCl_3_:MeOH (1:1) followed by 30 mL of CHCl_3_:MeOH (1:2). The combined aqueous phase was transferred to a 300 kDa dialysis tubing (31 mm × 200 mm, Biotech CE) and dialyzed against 10 L H_2_O for 36 h, refreshing H_2_O every 12 h. The residual liquid in the membrane was collected and lyophilized to yield a white fluffy powder (14 mg) that formed a gel-like consistency upon rehydration.

### Sample Preparation of Labeled *O*-Glycans and Dextran Ladder

Mucins (200 μg) and fetuin (500 μg) were dissolved in a 12.8 M ammonium carbamate solution (1 μg/μL) and incubated at 60 °C for 20 h. In order to remove residual proteins and peptides and prevent clogging of SPE cartridges, the samples were transferred to 3 kDa Amicon centrifugal filters and centrifuged at 14,000*g* for 30 min. 450 μL of water was added to the filters, and the samples were centrifuged again at 14,000*g* for 30 min. The released *O*-glycans present in the filtrate were enriched on Hypersep Hypercarb SPE cartridges and dried via vacuum centrifugation. Afterward, dextran *M*_w_ 1000 (100 μg) and *O*-glycans were dissolved in 100 μL of water each. 90 μL of 2 M procainamide hydrochloride in DMSO/acetic acid (8:1) and 120 μL of 3.2 M sodium cyanoborohydride in H_2_O were added. The mixture was incubated at 65 °C for 2 h. The glycans were precipitated by addition of acetone (4 mL). The labeled glycans were purified using Discovery Glycan SPE tubes and dried via vacuum centrifugation. The labeled *O*-glycans were dissolved in 25 μL of ACN/DMF/H_2_O (2:1:1). The labeled dextran *M*_w_ 1000 was dissolved in 100 μL of ACN/DMF/H_2_O (2:1:1).

### *O*-Glycan Analysis Using LC-FLD and LC–MS

LC-FLD experiments were performed using an Azura HPLC system (Knauer, Germany) equipped with a Dionex UltiMate 3000 fluorescence detector (Thermo Fisher Scientific). After separation, ProA-labeled glycans were analyzed using fluorescence detection (λ_ex_ = 310 nm, λ_em_ = 370 nm). The injection volume was 6 μL. LC–MS experiments were performed using a SYNAPT G2-Si spectrometer (Waters, U.K.) equipped with an Acquity UPLC system (Waters, U.K.). After separation, ProA-labeled glycans were ionized in ESI positive ion mode with a capillary voltage of 3.2 kV, and a source temperature of 150 °C. The mass range was set to *m*/*z* 400–2000. The injection volume was 7.5 μL. For LC-FLD and LC–MS experiments, the glycans were separated using an Acquity UPLC BEH amide column (130 Å, 1.7 μm, 2.1 mm × 150 mm, Waters, U.K.). The column oven was set to 65 °C, and the flow rate was set to 0.4 mL/min. Solvent A was 50 mM ammonium formate adjusted to pH 4.4, and solvent B was acetonitrile. Glycans were analyzed using an isocratic gradient at 90% B for 5 min, followed by a linear gradient of 90–60% B from 5 to 105 min. LC-FLD data was processed using Chromeleon (version 7.2.10, Thermo Fisher Scientific). LC–MS data was processed using MassLynx (version 4.1, Waters, U.K.) and MZmine 3.^42^ The retention times of GU from 2 to 11 derived from the ProA-labeled dextran ladder were used to generate retention time calibration curves via fifth-order polynomial regression of retention time vs log(GU).

## Results and Discussion

**Figure 1:**
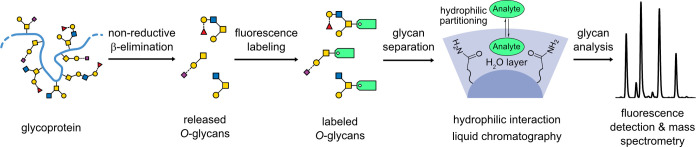
Illustration of the workflow from sample preparation to *O*-glycan analysis. The glycans are released from glycoproteins by nonreductive β-elimination, followed by tagging with a fluorescence label. The labeled *O*-glycans are separated using hydrophilic liquid interaction chromatography and analyzed by fluorescence detection and mass spectrometry.

A workflow including *O*-glycan labeling and analysis using HILIC separation coupled with fluorescence and mass spectrometry detection was developed (Figure 1). Generating free reducing end sugars, the glycans are released from the glycoproteins by nonreductive β-elimination using ammonium carbamate, which has shown great potential for quantification in the past.^43^ After two brief cleanup steps, the released glycans are fluorescently labeled at the reducing end of the core GalNAc. In this study, we chose the ProA label, which has a higher fluorescence intensity and better ionization efficiency than other commonly used fluorescent labels.^37^ Subsequently, the ProA-labeled *O*-glycans are separated by HILIC and detected by FLD and MS. In every sequence, we included a run of a ProA-labeled dextran ladder for retention time calibration.

**Figure 2:**
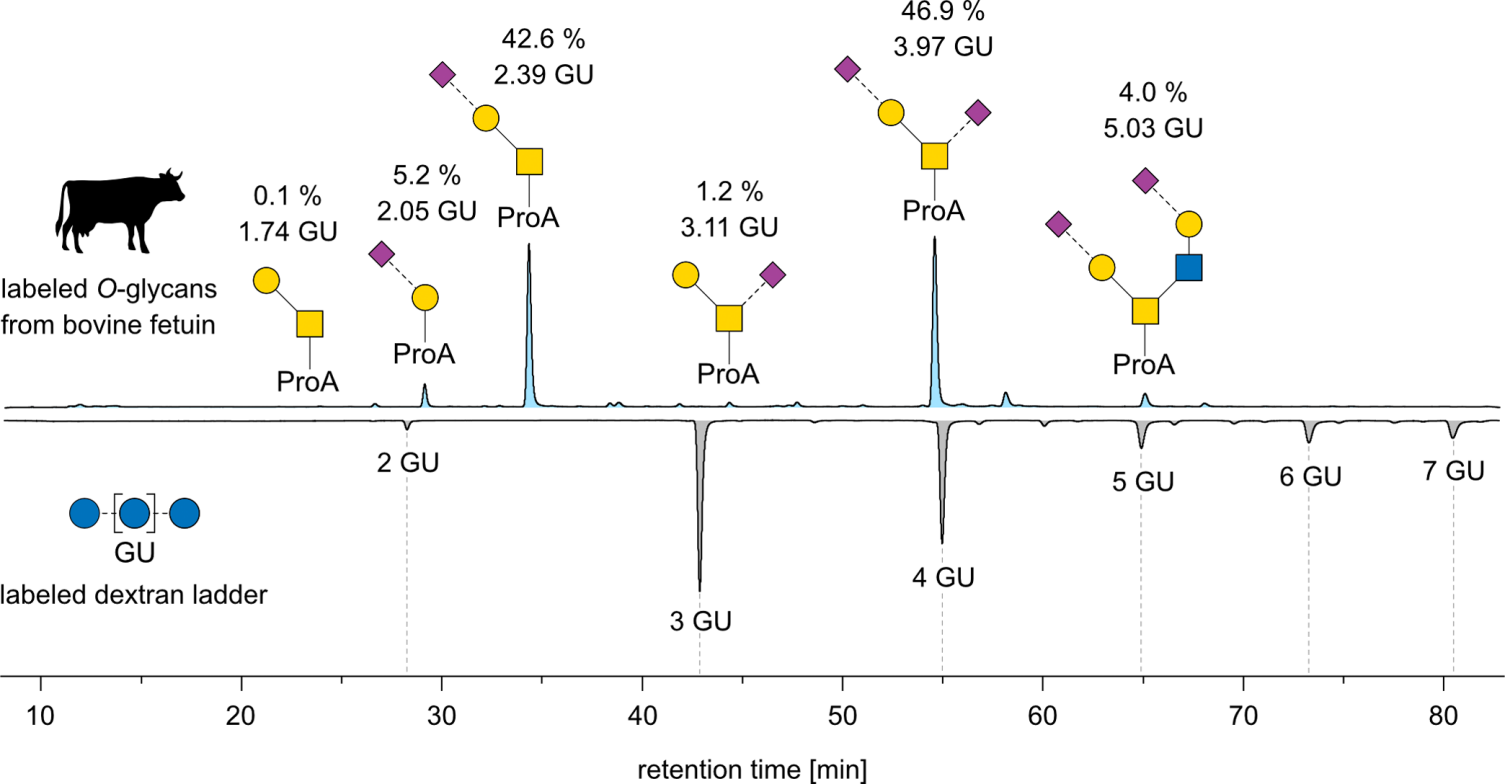
Chromatograms of released ProA-labeled *O*-glycans from bovine fetuin (top) and ProA-labeled dextran ladder (bottom). The chromatograms were recorded using fluorescence detection and the assignment of the peaks were performed by mass spectrometry. The assigned *O*-glycans are depicted using the symbol nomenclature for glycans (SNFG).^44^ The relative abundance and GU value of each *O*-glycan are noted above the structures.

Our method was first developed and tested on fetuin extracted from bovine fetal serum. This glycoprotein carries multiple *N*- and *O*-glycosylation sites and is a well-characterized model glycoprotein. Fetuin has a relatively simple *O*-glycan profile that consists of only six oligosaccharides.^38^Figure 2 shows the FLD chromatogram of ProA-labeled *O*-glycans from bovine fetuin. The six distinct peaks corresponding to *O*-glycan structures were assigned by mass spectrometry. The data show that the method is capable of releasing, labeling, separating, and identifying all *O*-glycans in this simple mixture. Relative abundances of the individual structures show that nearly all of the *O*-glycans in bovine fetuin carry one or two sialic acids. Other common modifications like fucosylation or sulfation have not been found. The third and fifth structures contribute to almost 90% of the *O*-glycan profile, while the rest is distributed across the four remaining structures. Due to the characteristic absence of GalNAc, the second structure at 2.05 GU was identified as a “peeling product” generated during the β-elimination process. This product accounts for 5% of the total profile, which is acceptable for a chemical *O*-glycan release. The relative abundance of each structure determined by MS can be found in Table S1. Comparison of FLD and MS data shows significant differences in the relative abundances of the individual glycans. The doubly sialylated structures are strongly decreased in terms of relative abundance, while the non- and monosialylated species are significantly increased, which is caused by different ionization behaviors due to the amphoteric nature of the labeled glycans and the instability of sialic acid moieties during and after the ionization process. These deviations demonstrate the benefit of *O*-glycan quantification by FLD in solution.

Generally, the relative quantification shows that every glycan carries an average of 1.5 sialic acids, which introduces a significant number of negative charges into the protein. The corresponding GU units above the identified *O*-glycan structures were calculated based on the retention times of the ProA-labeled dextran ladder that is plotted below. In order to test the reproducibility of the GU values, the measurements were performed again on a second identical HPLC system. The GU values reported in Table S1 show a difference of ≤0.04 GU, which demonstrates a good repeatability of the experiments. Comparison of the GU value to the actual number of monosaccharides of the *O*-glycan structures shows mixed results. While the GU values of the fourth and fifth structures at GU 3.11 and 3.97 seem to correlate well with the number of monosaccharide units, the GU value significantly underestimates the number of monosaccharides for the third and sixth structures at GU 2.39 and 5.03. However, the mismatch of the GU values and the number of monosaccharide units in *O*-glycans is expected because dextran is a linear chain of glucose units, while *O*-glycans are often branched and composed of different monosaccharide units such as galactose (Gal), fucose (Fuc), *N*-acetylneuraminic acid (Neu5Ac), *N*-glycolylneuraminic acid (Neu5Gc), and different *N*-acetylhexosamines (HexNAc).^44^ Comparison of the trisaccharide isomers at GU values of 2.39 and 3.11 further reveals that even subtle changes in the connectivity of the monosaccharide unit can have a major impact on the retention time. GU values are therefore only a rough estimate of the number of monosaccharide units in *O*-glycans.

Despite their diversity, GU values are powerful reference values to simplify the *O*-glycan structural assignment. From our set of identified *O*-glycans, we observed two general trends: (1) With an increasing number of monosaccharides, the overall polarity of the *O*-glycan is increased, which enhances the retention in HILIC. (2) The addition of specific structural features increases the GU value in the order Fuc < Neu5Ac < Neu5Gc. These trends are in good agreement with the retention behavior generally observed for *N*-glycans on HILIC phases.^45^

**Figure 3:**
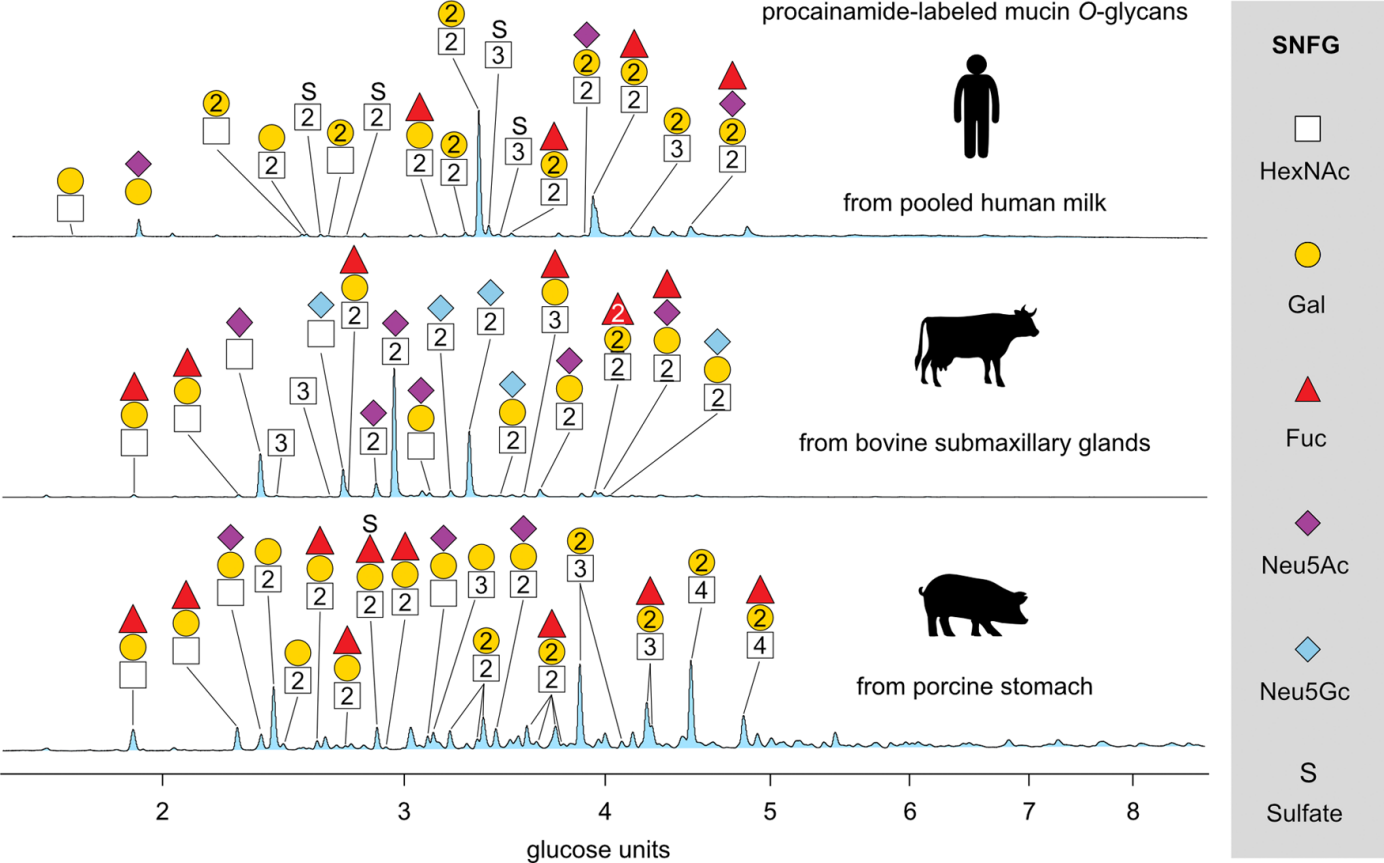
Chromatograms of ProA-labeled *O*-glycans from human milk mucins (HMM), bovine submaxillary mucins (BSM), and porcine gastric mucins (PGM) recorded by fluorescence detection. The retention time was converted to the glucose unit (GU) scale. All *O*-glycan compositions assigned by mass spectrometry are depicted using the SNFG.^44^

**Figure 4:**
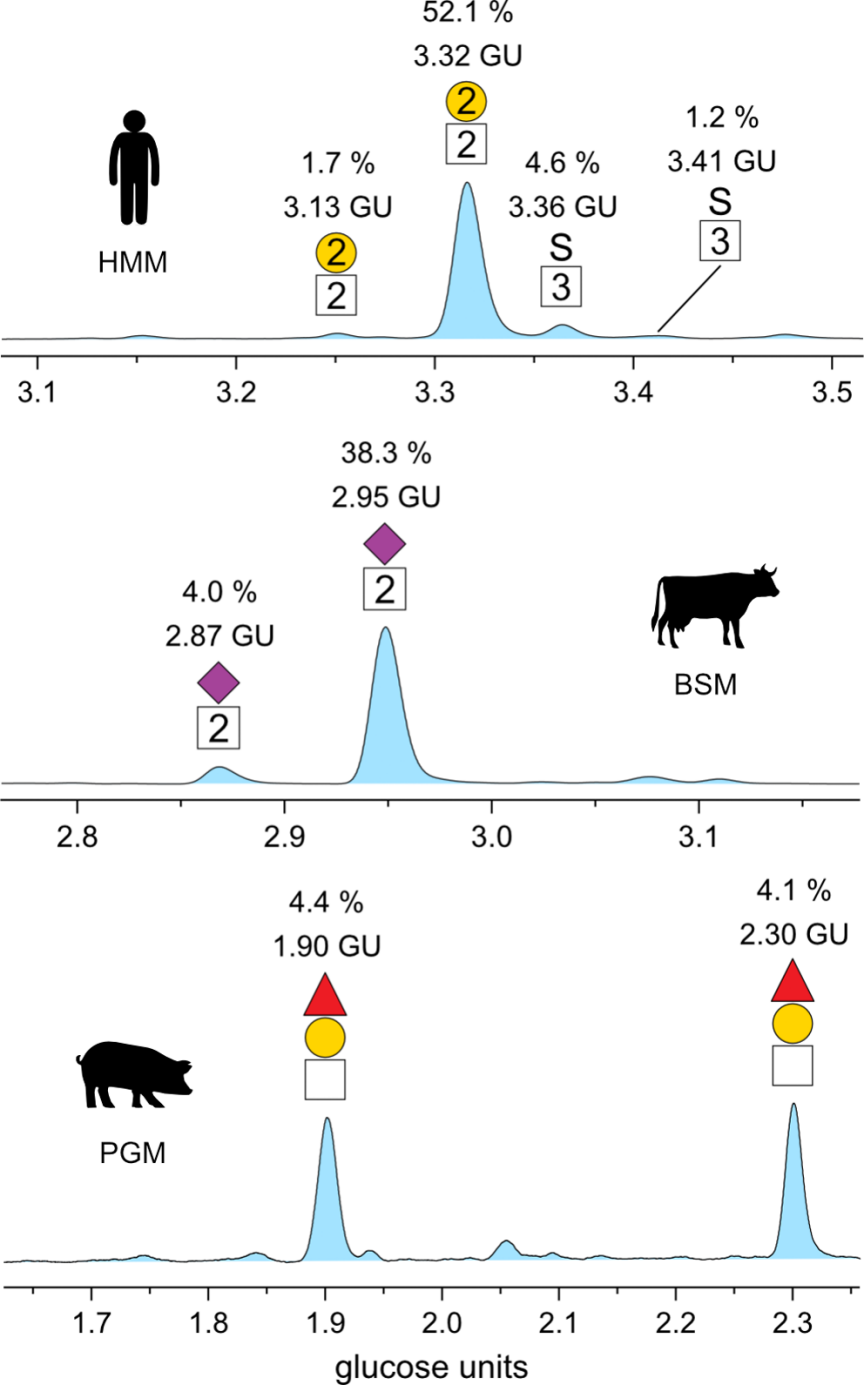
Separation of selected *O*-glycans isomers in the chromatograms of human milk mucins (HMM, top), bovine submaxillary mucins (BSM, middle), and porcine gastric mucins (PGM, bottom). The relative abundance and GU value of each *O*-glycan isomer are noted above the structures.

To test the workflow on more complex mucin-type *O*-glycan samples, it was applied to membrane-bound and secreted mucins. Samples from three different types of tissues and mammals were selected to comprehensively cover the high structural diversity of *O*-glycans. The three samples include mucins from pooled human milk (mainly hMUC1, and hMUC4),^46^ bovine submaxillary glands (mainly bMUC19)^47^ and porcine stomach (mainly pMUC5AC, pMUC5B, and pMUC6).^48^Figure 3 shows the chromatograms of labeled *O*-glycans from human milk mucins (HMM), bovine submaxillary mucins (BSM), and porcine gastric mucins (PGM) recorded by HILIC-FLD. The compositions of the most abundant structures were assigned by MS and are indicated on the chromatograms. All chromatogram peaks were integrated, and the relative abundance for each *O*-glycan structure is reported in Table S1, together with their corresponding GU values. While all three traces show high complexity, the chromatogram of PGM appears to be the most congested. Assignment of the most prominent peaks and comparison of the profiles reveal vastly different combinations of *O*-glycans. In total, 37 compositions were identified in the mucin samples. Multiple peaks in the chromatograms were found to be caused by glycans with the same composition, which indicated the separation of several *O*-glycan isomers. Including isomers, different numbers of individual *O*-glycans were identified for HMM (14 compositions and 21 structures), BSM (20 compositions and 27 structures), and PGM (14 compositions and 28 structures). For BSM and PGM, the numbers of identified *O*-glycan structures are comparable with recent LC_PGC_-MS studies on the corresponding *O*-glycan alditols.^28,49^ As a consequence and considering the batch-to-batch variation of the biological source material, it can be concluded that this method is capable of detecting most of the structures with moderate abundance. Based on the signal-to-noise ratios of the low abundant peaks in the fluorescence chromatogram (approximately 25–35), a decrease of the amount of starting material by a factor of 10 without the loss of minor peaks should be achievable. Nevertheless, preparative scale studies show that additional *O*-glycan structures with very low abundances can be found in BSM.^50,51^ Therefore, it cannot be ruled out that some minor *O*-glycans are not detected by this method. Dividing the number of individual structures by the number of compositions yields the average number of isomers per composition. With an average of two isomers per glycan composition, PGM shows the highest isomer content. HMM and BSM *O*-glycans show fewer isomers, with an average of approximately 1.4 isomers per composition. A more detailed and exemplary representation of the isomer separation can be found in Figure 4. The four selected examples taken from the chromatograms of the three mucin samples show that multiple peaks can be found for the same composition of sulfated, sialylated, and fucosylated *O*-glycan structures, as well as for unmodified structures consisting of only Gal and HexNAc units. These observations suggest that *O*-glycan isomer separation in HILIC occurs independently of the glycan composition and the presence of glycan features. However, the difference in retention for the individual isomers varies in every case. While the fucosylated isomers (Gal_1_HexNAc_1_Fuc_1_) show a far separation with a difference of 0.4 GU, the sulfated isomers (HexNAc_3_S_1_) have a very similar retention with a GU difference of only 0.05 GUs. The difference in retention time/GU might give a hint about the structural similarity of the isomers. Furthermore, the detailed separation allows the relative quantification of the individual *O*-glycan isomers in the sample and offers the opportunity to determine their ratios. The fucosylated isomers (Gal_1_HexNAc_1_Fuc_1_) in our example show similar relative abundances and a ratio of approximately 1:1. A ratio of 4:1 is observed for the sulfated isomers (HexNAc_3_S_1_) and a 1:10 ratio for the sialylated isomers (HexNAc_2_Neu5Ac_1_). The largest difference is observed for the Gal_2_HexNAc_2_ isomers with a ratio of 1:31.

**Figure 5:**
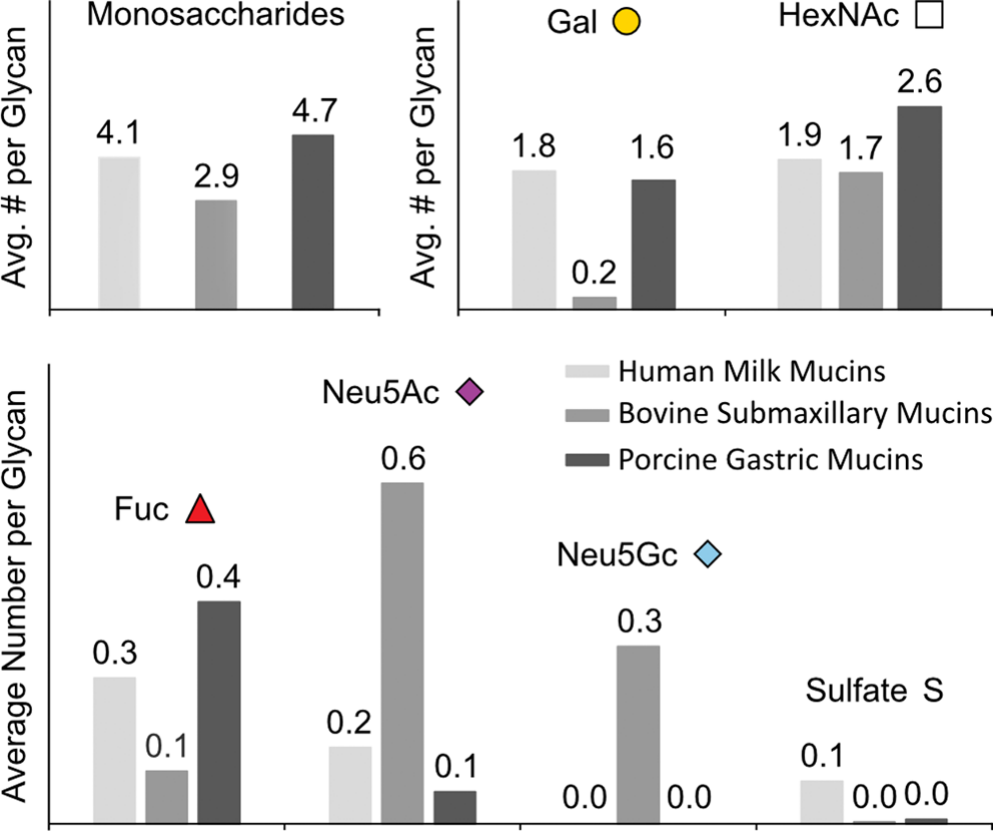
Bar charts representing the average number of monosaccharide units per glycan (top left), Gal and HexNAc units per glycan (top right), and Fuc, Neu5Ac, and Neu5Gc units and sulfates per glycan (bottom) for human milk mucins (HMM, light gray), bovine submaxillary mucins (BSM, middle gray), and porcine gastric mucins (PGM, dark gray).

With the high number of individual *O*-glycans per sample, simultaneous visualization of all relative abundances becomes challenging. In order to simplify the interpretation and make quantitative statements on the samples’ glycosylation, we determined the average number of total monosaccharides and specific features per glycan, using the relative peak areas and the compositions of the corresponding *O*-glycans (Figure 5). The first bar chart represents the mean number of monosaccharides per glycan in each sample. BSM *O*-glycans show the lowest number of monosaccharides, with an average of about three units per glycan. The *O*-glycans of HMM show an average size of a tetrasaccharide. With an average of almost five monosaccharides per glycan, the highest number of monomeric units is observed in PGM. This observation correlates well with information about the average number of isomers per glycan composition. With an increasing average number of monosaccharides in PGM, the number of isomers is likely to increase, as well.

Next, the average number of Gal and HexNAc units per glycan in the three different mucin samples is shown. On average, HMM *O*-glycans are composed of almost equal numbers of Gal and HexNAc units per glycan. For both Gal and HexNAc, an average of two units per glycan can be observed. These numbers suggest that HMM *O*-glycans are mostly extended from a core 1 structure (Gal_1_GalNAc_1_), which provides equal numbers of Gal and HexNAc. While the number of HexNAc units per glycan is almost identical in BSM *O*-glycans, galactosylation is significantly lower. Here, we can hypothesize that BSM *O*-glycans are extended mostly from core 3 (GlcNAc_1_GalNAc_1_) and core 4 (GlcNAc_2_GalNAc_1_), providing an explanation for the high content of HexNAc. For PGM, a different trend can be observed. While the average number of galactose units per glycan is almost identical to HMM, the number of HexNAc units tends to be significantly higher compared to BSM and HMM. This can correspond to glycan structures in PGM *O*-glycans that are mostly elongated from a core 2 structure (GlcNAc_1_Gal_1_GalNAc_1_). This correlates well with previous reports on PGM *O*-glycosylation profiles determined by LC–MS, showing that PGM *O*-glycans are dominated by neutral core 2 glycans.^52^

Finally, we present the abundance of specific *O*-glycan features such as Fuc, Neu5Ac, Neu5Gc, and sulfate groups in the sample set. The highest number of fucose units per glycan can be observed in PGM *O*-glycans, which suggests that on average about 40% of the total *O*-glycans carry one fucose unit. The lowest fucosylation is observed in BSM, where on average only every tenth of the *O*-glycan appears to be fucosylated. Looking at sialylation, the highest number of Neu5Ac units can be found for BSM. About 60% of the overall *O*-glycans in BSM appear to carry one Neu5Ac. With a 6-fold lower value, PGM *O*-glycans carry the least Neu5Ac units per glycan. Neu5Gc units were found in only one of the three samples. In BSM, 30% of the *O*-glycans carry a Neu5Gc unit. Combining the number of sialic acids (Neu5Ac and Neu5Gc) in the BSM, we can conclude that approximately one sialic acid unit per *O*-glycan can be observed. Compared to all other glycan features, the degree of sulfation is very low in all samples. In BSM and PGM, only 0.3 and 0.8% of the *O*-glycan structures were found to carry a sulfate group. The highest average number of sulfates per glycan was found for HMM, where one out of ten *O*-glycans carries one sulfate group.

Overall, it can be summarized that the sugar-coating of BSM is comparably short and, at the same time, highly negatively charged. On the other hand, PGM carries a wide distribution of *O*-glycan structures, which are comparably neutral and rich in fucose. The opposing glycan profiles of BSM and PGM lead to different lubricating and viscoelastic properties as well as antiviral properties.^53,54^ HMM also carries mostly neutral and fucosylated *O*-glycans. However, due to the comparably high degree of sulfation and presence of 2-fold more sialic acids, HMM presents more negative charge carriers on its carbohydrate shell compared to PGM.

## Conclusions

Due to their structural complexity, the analysis of mucin-type *O*-glycans usually requires hyphenation of multiple analytical techniques.^22,25^ Mostly based on mass spectrometry, these techniques constitute efficient tools for qualitative assessment of *O*-glycosylation but are not strictly quantitative. In this study, we developed a new workflow to introduce fluorescent labeling and detection into the field of mucin *O*-glycomics. We demonstrate the utility of this method for the direct quantification of mucin-type *O*-glycan profiles from diverse types of tissues and mammals. As a proof of principle, we analyzed *O*-glycans from pooled human milk, bovine submaxillary glands, and porcine stomach and highlighted the particularities of their individual glycosylation profiles by comparing the characteristic structural elements. This includes the content of specific *O*-glycan features such as fucoses, sialic acids, sulfates, galactoses, and *N*-acetylhexosamines and provides an overview of the average size of the glycans present in the samples. The data show that bovine submaxillary mucin *O*-glycans are heavily sialylated by Neu5Ac and Neu5Gc units, while the *O*-glycan profiles of human milk and porcine gastric mucins are richer in neutral features like HexNAc, galactose, and fucose. In order to provide a simple approach for the retention time calibration in *O*-glycomics, we utilized the concept of fluorescently labeled dextran ladders and discussed their scope and potential. Based on the obtained data, we created a glucose unit library for all identified *O*-glycans, which can be used for putative assignments. The workflow developed in this study offers great potential for the field of glycomics, as it allows for the quantification of individual *O*-glycans in mucins and increases the reproducibility and comparability of *O*-glycomics data. The dense *O*-glycosylation of mucins gives its defensive properties to mucus and is significantly altered in diseases such as cystic fibrosis, chronic obstructive pulmonary diseases, or cancer. This robust quantification method provides a powerful tool to better characterize the glycosylation-dependent interactions of the mucus with pathogens, study disease progression and potentially serve diagnostic purposes.

## Supporting Information Available

Identified *O*-glycan compositions with retention times, GU values, and relative abundances (Table S1) (XLSX)

## Abbreviations

HMM: human milk mucins
BSM: bovine submaxillary mucins
PGM: porcine gastric mucins
GU: glucose unit
FLD: fluorescence detection
MS: mass spectrometry
HILIC: hydrophilic interaction liquid chromatography
ProA: procainamide
